# Analytical Capability of High-Time Resolution-Multiple Collector-Inductively Coupled Plasma-Mass Spectrometry for the Elemental and Isotopic Analysis of Metal Nanoparticles

**DOI:** 10.5702/massspectrometry.A0085

**Published:** 2020-06-12

**Authors:** Takafumi Hirata, Shuji Yamashita, Mirai Ishida, Toshihiro Suzuki

**Affiliations:** 1 Geochemical Research Center, The University of Tokyo; 2 Technology and Intellectual Property HQ, TDK

**Keywords:** Os isotopic, nanoparticles, MC-ICP-MS, high-time resolution data acquisition

## Abstract

We measured the Re/Os (^185^Re/^188^Os) and ^187^Os/^188^Os ratios from nanoparticles (NPs) using a multiple collector-inductively coupled plasma-mass spectrometer equipped with high-time resolution ion counters (HTR-MC-ICP-MS). Using the HTR-MC-ICP-MS system developed in this study, the simultaneous data acquisition of four isotopes was possible with a time resolution of up to 10 μs. This permits the quantitative analysis of four isotopes to be carried out from transient signals (*e.g.*, <0.6 ms) emanating from the NPs. Iridium–Osmium NPs were produced from a naturally occurring Ir–Os alloy (ruthenosmiridium from Hokkaido, Japan; osmiridium from British Columbia, Canada; iridosmine from the Urals region of Russia) through a laser ablation technique, and the resulting nanoparticles were collected by bubbling water through a suspension. The ^187^Os/^188^Os ratios for individual NPs varied significantly, mainly due to the counting statistics of the ^187^Os and ^188^Os signals. Despite the large variation in the measured ratios, the resulting ^187^Os/^188^Os ratios for three Ir–Os bearing minerals, were 0.121±0.013 for Hokkaido, 0.110±0.012 for British Columbia, and 0.122±0.020 for the Urals, and these values were in agreement with the ratios obtained by the conventional laser ablation-MC-ICP-MS technique. The data obtained here provides a clear demonstration that the HTR-MC-ICP-MS technique can become a powerful tool for monitoring elemental and isotope ratios from NPs of multiple components.

## INTRODUCTION

Mass spectrometry utilising an inductively coupled plasma as an ion source (ICP-MS) was used to measure both the sizes and number concentrations of nanoparticles (NPs) in aqueous solutions.^1–3)^ The typical settling time for mass switching (switching delay time), used in the conventional quadrupole-based MS, is about 1 ms, which is comparably longer than the time duration of a signal event obtained from a single NP (*i.e.*, 0.2 to 0.6 ms).^4)^ This indicates that only a single element (isotope) can be monitored using the conventional ICP-MS system (*e.g.*, single particle-ICP-MS: spICP-MS).^5–9)^ Although fast size distribution analysis of NPs was achieved, analytical restriction still remains, since the system is not capable of monitoring the elemental composition or the isotope ratios of the constituent elements.

Elemental analysis for multicomponent NPs (*e.g.*, metallic alloy or non-metallic compounds) or structures (*e.g.*, core-shell or polarized structures) are increasingly needed. An ICP-based mass spectrometer using a time of flight type mass spectrometer (ICP-TOF-MS) was reported to be able to monitor multiple elements (isotopes) of NPs.^10–12)^ With the ICP-TOF-MS system, multiple elements or isotopes can be monitored from the transient signals obtained from NPs.^13)^ Despite its ability to obtain elemental ratio data from single NPs, it should be noted that the time efficiency of signal integration (*i.e.*, duty cycle of the data acquisition) achieved by the ICP-TOF-MS was significantly lower than that for a spICP-MS system. This suggests that the uncertainties in the size distribution analysis obtained with the ICP-TOF-MS system could be greater than the corresponding values for a conventional spICP-MS system.

Another effective method for detecting multiple elements or isotopes from single NPs is to use magnetic sector based ICP-MS instruments equipped with a multiple collector (MC) system. The simultaneous detection of multiple elements or isotopes can be made without lowering the time efficiencies for the signal integrations. To obtain quantitative signal intensity data from small ion sizes (*e.g.*, 10–1,000 ions) and transient signals (*e.g.*, time duration of 0.2–0.6 ms), high-gain ion detectors, such as electron multipliers^14)^ or Daly ion detectors^15)^ must be used. Using the multiple collector system with high-gain ion detectors, the signal intensities of multiple elements (isotopes) can be monitored with time integration efficiency better than that achieved by the ICP-TOF-MS system.

Isotopic data of the constituent elements can also provide key information concerning the origin and formation sequence of the NPs. Changes in isotopic abundance, due to the radioactive decay of parent nuclides, have been widely used as geochronology and isotope tracers. Among the isotope systematics, a ^187^Re–^187^Os decay system based on the radioactive decay of ^187^Re with a half-life of about 40 Byrs, can be used to identify the timing of crust/mantle separation, or metal/silicate segregation of planets or the parent body of a meteorite.^16–19)^ Osmium is enriched in metallic nuggets, or certain Os-bearing minerals, such as iridosmine, osmiridium, rutheosmiridium, and these minerals contain virtually no Re, suggesting that the contribution of isobaric interference on ^187^Re on ^187^Os would be negligible.^16–18)^ More importantly, recent studies revealed that the ^187^Os/^188^Os ratios reflect the nucleosynthetic processes of heavy elements.^20–24)^ Based on the elemental and isotopic signatures derived from individual NPs, information such as the origin of the elements (Fe to U), the transport mechanism of NPs, and the formation processes of our Solar system, can be obtained. Several pioneering research studies revealed that the Os is present as metallic alloy with Ir, and some other platinum group elements (PGE), whrease Re was present in the form of separated grains associated with other chalcophile elements such as S, or Mo, obviating the larger contribution of mass spectrometric interferences on ^187^Os by ^187^Re, or *vice versa*.^25,26)^ However, because of the limited sizes of the these metallic grains, which are μm-sized or nm-sized metallic nuggets or particles, the elemental and isotopic analysis of the NPs has not progressed, mainly due to analytical difficulties. Because of the analytical difficulty in obtaining the Os isotope data from individual micro-grains, precise isotope ratio measurements of Os from bulk chondrites have been used as acceptable.^27–29)^ However, the Os isotope data obtained from bulk chondrites are averaged values obtained from the analysis of large numbers of small grains. To overcome this, we developed a new analytical technique for measuring ^187^Os/^188^Os ratios from Os–Ir bearing NPs of various sizes.

In this study, we interfaced a four-channel high-time resolution amplifier system to an MC-ICP-MS equipped with a multiple ion counting system. With high-time resolution ion counters, the signal intensities of up to four elements (isotopes) can be quantitatively monitored with a time resolution of up to 10 μs, and therefore, quantitative signal intensity data can be obtained from single signal events with short time durations, thus enabling us to monitor the elemental or isotope ratios of the NPs. Nevertheless, the isobaric interference on ^187^Os by ^187^Re, and ^190^Os by ^190^Pt, and ^192^Os by ^192^Pt, must be taken into account. Since Re (chalcophile element) has different geochemical features from Os and Ir (siderophile elements), the Re/Os abundance ratios for Ir–Os bearing minerals would be lower than 10^−4^, hence the contribution of the isobaric interferences by Re would be negligibly small.^16–19,25,26)^ In contrast, the geochemical characteristics of Pt are similar to Os, and isobaric interferences by Pt can cause systematic errors in the measured Os isotope ratios. Due to both the limited number of isotopes (*i.e.*, four isotopes) and mass dispersion of the mass spectrometer, we were not able to monitor Pt isotopes using the present system setup. Hence, correction of the mass discrimination effect was corrected by normalising the ^189^Os/^188^Os ratio.

The application of the present HTR-MC-ICP-MS technique can be further extended to the precise isotopic ratio analyses of trace-elements. The measured count rates cannot reflect the true count rate of the ion signals. This is especially true in the case of transient signals. Data acquisition with a poorer time resolution (*i.e.*, a longer dwell time) can lower the measured count rate, resulting in under estimation of the counting loss due to dead time. Both the high-time resolution and the signal-output linearity of the ion counters are still a key issue in terms of improving data quality in the isotope ratio analysis.

## EXPERIMENTAL

### Instrumentation

**Figure 1:**
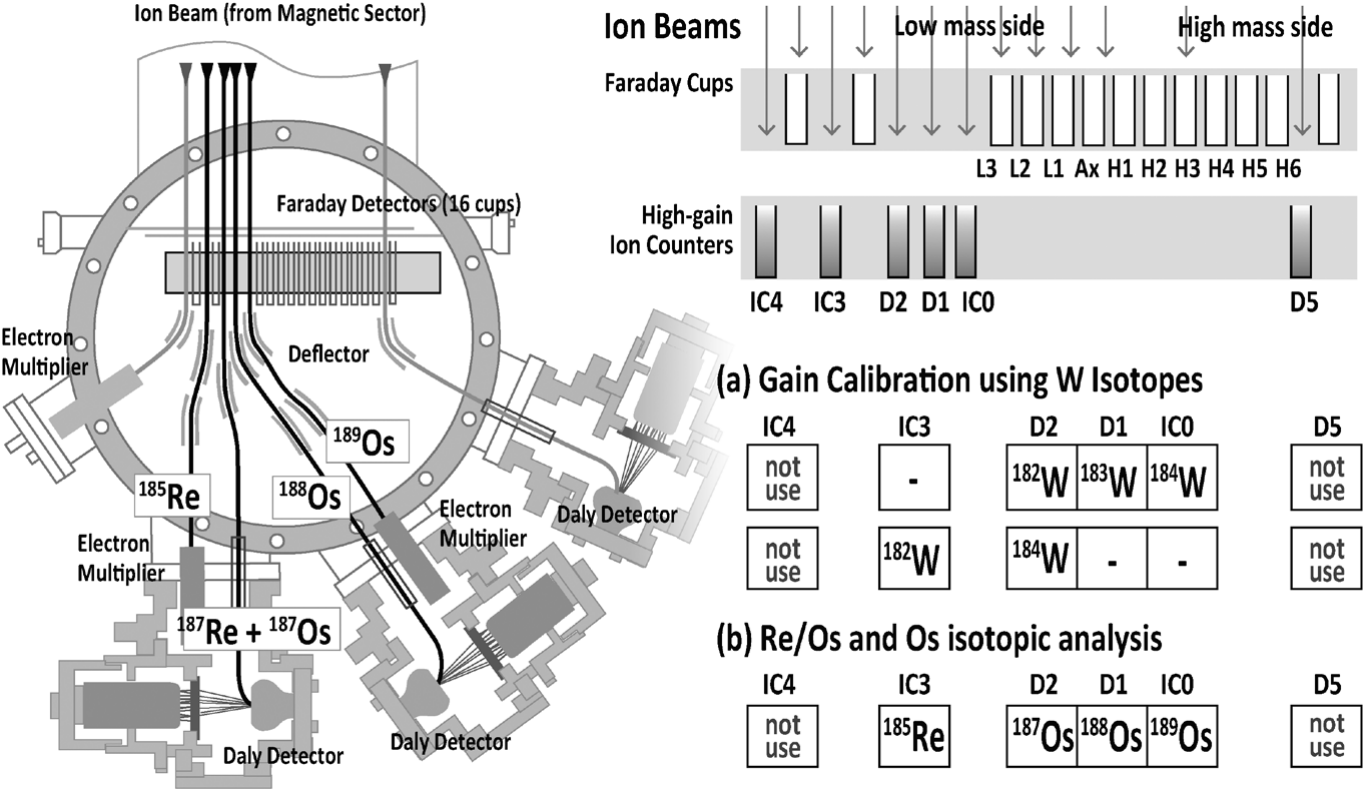
Fig. 1. Detector arrangements for the simultaneous detection of four isotopes from transient signals emanating from nanoparticles.

The ICP-MS instrument used in this study was a multiple collector ICP-MS (Nu Plasma 2, Nu Instruments, Wrexham, UK). The system is capable of measuring ion currents of a total of 22 isotopes using 16 Faraday detectors and six high-gain ion detectors (three electron multipliers and three Daly ion detectors).^28,29)^ The Faraday detectors were used for the isotope analysis of Os using the conventional laser ablation sampling technique. Hence, the detector arrangement is Faraday L3 for ^185^Re, L2 for ^186^(W+Os), L1 for ^187^(Re+Os), Axial for ^188^Os, H1 for ^189^Os, H2 for ^190^(Os+Pt), H3 for ^191^Ir, H4 for ^192^(Os+Pt), H5 for ^193^Ir, and H6 for ^194^Pt (Fig. 1).

High-gain ion detectors were used for the elemental and isotopic analysis of the NPs. Because the time response of the Faraday detector (*i.e.*, current monitoring) is not fast enough to keep up with the rapid changes in the signal intensities emanating from the NPs.^30)^ The quantitative signal intensity data from transient signals were detected by high-gain ion detectors combined with high-time resolution data acquisition systems (we refer to this system as high-time resolution-MC-ICP-MS: HTR-MC-ICP-MS). Four high-time resolution amplifiers were applied to the MC-ICP-MS system, and a wide range of the dwell times from 1 s down to 10 μs can be selected. In this study, dwell times of 30 μs (*i.e.*, sampling rate of *ca.* 33 kHz) were used for the four ion counters in all of the measurements. The resulting time resolution is sufficiently rapid to permit the rapid changes in the signal intensities for the transient signals obtained from the NPs to be followed.^31)^

The dead time of the ion counters were measured separately by monitoring the isotope ratios obtained by a combination of high-gain ion detectors and Faraday detectors. Details of procedures for the calibration of dead time have been reported in our previous paper.^14)^

### Corrections of detector gain

Among the six high-gain ion detectors, four ion detectors of various combinations were applied for the simultaneous detection of ion signals of W, Re, and Os isotopes from the NPs (Fig. 1). The gains of the high-gain ion detectors were dependent upon the high voltages applied to the multipliers and discrimination settings for individual ion counters. The relative difference in the detector gain was calibrated using ^182^W, ^183^W, and ^184^W isotopes. The gain calibration consists of two-sequential isotope ratio measurements using different combinations of two high-gain detectors: (1) ^183^W (Daly 1: D1)/^182^W (Daly 2: D2), and ^184^W (Electron Multiplier: EM0)/^182^W(D2) ratio measurement, and followed by (2) ^184^W (D2)/^182^W (EM3) ratio measurements (Fig. 1). The relative gains of the four high-gain ion detectors were calculated by normalizing the measured ^183^W/^182^W, and ^184^W/^182^W ratios being 0.5428 and 1.172, respectively, using a simple linear set up.^32)^ Details of the instrumentation and operational settings were listed in Table 1.

**Table 1:** Table 1. Instrumental and operational settings.

ICP-mass spectrometer	
Instrument	Nu Instruments NP2 MC-ICP-MS
RF power	1.3 kW
Nebuliser	MicroMist 200 (uptake rate 200 μL/min)
Spray chamber	Cyclonic (4°C)
Detection mode	Static
Interface	HS Interface (Dry mode)
Data acquisition	4 Channel High-Speed Pararell Signal Integrator
Monitored isotopes	
LA-ICP-MS analysis	^185^Re(F), ^186^Os(F), ^187^Os(F), ^188^Os(F)
	^189^Os(F), ^190^Os(F), ^191^Ir(F), ^192^Os(F)
Dwell time	0.2 s (TRA mode)
Data acquisition	60 s
Nanoparticles	
Gain calibration	Sequence 1: ^182^W (D2), ^183^W (D1), ^184^W (IC0)
	Sequence 2 : ^182^W (IC3), ^184^W (D2)
Os analysis	^185^Re (IC3), ^187^Os (D2), ^188^Os (D1), ^189^Os (IC0)
Dwell time	30 μs
Settling time	0
Dead time	16 ns for IC3, 8 ns for D2, 12 ns for D1, and 14 ns for IC0
Dead time correction	Non-extended model
Calibration	
Bias correction	
LA-ICP-MS analysis	^188^Os/^192^Os=0.3244 [17,33]
Correction law	Exponential law
Gain calibration	^183^W/^182^W=0.5428 [34]
	^184^W/^182^W=1.172 [34]
Laser ablation	
Laser	Frequency Quadrupled Yb:KGW Femtosecond Laser
Pulse duration	230 fs
Fluence	1 J/cm^2^
Repetition rate	1–2 Hz
Production of nanoparticles	
Laser	Frequency Quadrupled Yb:KGW Femtosecond Laser
Fluence	4–6 J/cm^2^
Repetition rate	1 kHz
Raster speed	10 μm/s
Sampling time	3 min
Carrier gas	He
Trap	Bubbling in 5 mL deionised water
Size calibration	60 nm Au NPs (nanoComposix)

### Data reduction

With the present system setup, signal intensity data for ^185^Re, ^187^(Re+Os), ^188^Os, and ^189^Os isotopes were simultaneously measured every 30 μs (dwell time) for 30 s. The resulting signal intensity profiles were then used to calculate the signal intensities (total counts of each isotope) of signal events using our in-house software “NanoQuant.” This software is used to calculate the total ion counts of the individual signal events. The signal events originating from the NPs were defined as continuous signal counts (>1 counts) for three time slices (*i.e.*, time duration of 90 μs). The total signal counts of the signal events were calculated by summing up the signal counts obtained for each time slice, hence the end of the signal events were defined by continuous signal counts of zero for three time slices (*i.e.*, a time duration of 90 μs). To avoid the duplicated integration of two or more signal events (mainly due to the overlap of the two or more signal events reaching the detector concurrently), the signal intensity profile of all events (signal intensity plotted against time) were visualized automatically by the NanoQuant software. Data showing potential overlaps of multiple signal events were not used in further calculations.

Corrections for counting loss due to dead time were made on individual signal counts based on a conventional non-extended protocol. For the isotope ratios, the signal intensity of the isotopes was calculated by integrating the total ion counts for individual signal event using the NanoQuant software. The resulting signal intensity data for each isotope were then used for the calculation of ^185^Re/^188^Os and ^187^Os/^188^Os isotope ratios.

### Background correction

The background count rate for ^185^Re, ^187^(Re+Os), and ^188^Os were all <20 cps. The signal count was less than 1 count per single data acquisition sequence when the dwell time was set to 30 μs. This suggests that the contribution of Os memory on the instrument and blank Os in the analysis solution will be almost identical to the electrical noise.

### Samples and sample preparation

For the Re/Os and Os isotope ratio measurements, three naturally occurring Ir–Os samples containing minerals from Japan, Canada, and Russia were used. Osmiridium and iridosmine is a natural alloy of osmium and iridium, with traces of other platinum-group metals, and ruthenosmiridium is a natural alloy of ruthenium, osmium, and iridium.

(a) Hokkaido (ruthenosmiridium): Ruthenosmiridium was defined as a new species of a natural alloy of Ru–Os–Ir.^35,36)^ In the chemical compositional diagram for minerals, the compositional field for ruthenosmiridium or rutheniridosmine was restricted by the revised nomenclature for alloys in which Ir was the dominant element.^37)^ The ruthenosmiridium samples from the Sorachi River, Hokkaido, Japan, were found as placers, and were considered to be derived from ultramafic rocks in the Kamuikotan metamorphic belt. The size of these samples were 1–2 mm.
(b) British Columbia (osmiridium): Osmiridium crystallizes in the isometric system and exhibits an iridium crystal lattice structure. Osmiridium occurs as an irregular shaped material and various sizes ranging from μm to mm scale, sometimes as concretions of grains and crystals are common.^38,39)^ The osmiridium samples from British Columbia, Canada, are associated with young ultramafic rocks in the northern part of the Garlock fault, and source of the Ir–Os bearing minerals could be the Tulameen mafic–ultramafic complex. The typical size of the sample is 0.05–0.5 mm.
(c) Urals (iridosmine): The placers of the Urals are associated with dunites in the Hercynian ophiolite belt. Since the precise isotopic data for iridosmine from the Urals Nevyansk were reported by Allègre and Luck,^19)^ and also by us,^40)^ this sample was used for the inter-laboratory comparison of the Os isotopic data. The typical size of the sample is about 0.5–0.8 mm.

**Figure 2:**
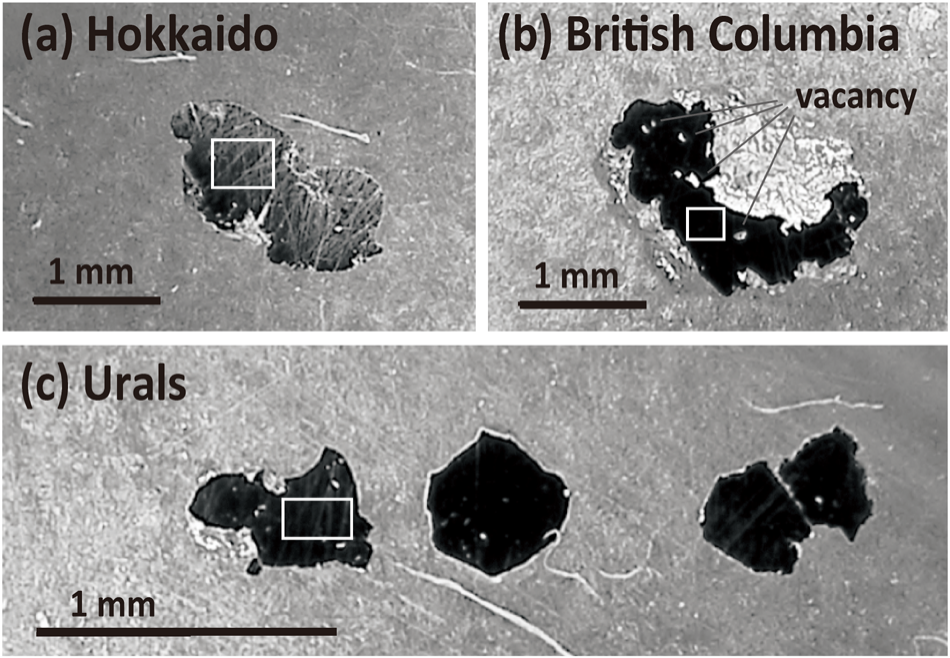
Fig. 2. Photographic images of three Ir–Os bearing minerals subsidized to Os isotopic analysis. Sampling areas were shown in white squares.

Sample grains used in the Os isotopic analyses were mounted using a resin (UK-M, PRESI, Eybens, France), and the sample surface was polished successively using #8000 polishing paper (Scotch Bright Polishing Sheet, 3M, St. Paul, MN, USA) and diamond paste (#10,000) to produce a flat surface. Photographic images of the sample are shown in Fig. 2.

NPs of the samples stated above were produced by laser ablation by using a 4HG (fourth harmonic generation) Yb:KGW femtosecond laser operating at a wavelength of 260 nm. A fluence of 4–8 J cm^−2^ and a repetition rate of 1 kHz were used. Laser ablation was carried out for 5 min by rastering the laser beam with a speed of 10 μm/s. The sampling areas are shown as white squares in Fig. 2. The laser induced sample aerosols were collected by bubbling air through a sample in 5 mL of deionized water. The number concentrations of the resulting NPs were about 10^5^–10^6^ particles/mL. Since higher number concentrations will induce overlaps of two or more signal events, the number concentrations of the NP solutions were adjusted to 10^5^ particles/mL using deionized water prior to the HTR-MC-ICP-MS measurements. The solutions were then introduced into the ICP using a Micromist nebulizer (Glass Expansion, Port Melbourne, Australia).

## RESULTS ANS DISCUSSION

### Os isotope ratio analysis using conventional LA-ICP-MS

Prior to the isotopic ratio measurements on the NPs, reference Os isotope ratios (^186^Os/^188^Os, ^187^Os/^188^Os, ^189^Os/^188^Os, ^190^Os/^188^Os, and ^192^Os/^188^Os) for the three Ir–Os bearing minerals were separately measured using the conventional laser ablation-MC-ICP-MS technique (LA-MC-ICP-MS) using the same laser ablation system for NP generation. The ion currents for ^185^Re, ^186^Os, ^187^(Re+Os), ^188^Os, ^189^Os, ^190^(Os+Pt), ^191^Ir, ^192^(Os+Pt), and ^193^Ir, isotopes were measured by Faraday detectors equipped with an amplifier using a negative feedback register of 10^11^ ohm. The ^190^Os and ^192^Os isotopes might be interfered by the ^190^Pt and ^192^Pt isobars, respectively, and therefore, the mass discrimination effect found in the MC-ICP-MS system was corrected by normalizing the ^189^Os/^188^Os to be 1.216 using the exponential law.^17,32)^ Hence, no correction of the isobaric interferences on ^190^Os and ^192^Os by ^190^Pt and ^192^Pt respectively were made in this study. The analytical session consisted of 30 s on-peak baseline measurements without laser ablation, followed by a 60 s data acquisition with laser ablation using a time-resolved analysis (TRA) mode. A dwell time of 0.2 s was adopted. After the subtraction of the baseline signals, signal intensity data of ^192^(Os+Pt) that was >1 V were used for calculating the isotope ratios. The resulting Re/Os and Os isotope ratios for three Ir–Os bearing minerals are summarised in Table 2. The measured ^185^Re/^188^Os ratios for Hokkaido, British Columbia, and Urals samples were <6.5×10^−5^, <2.5×10^−4^, and 10^−4^, respectively. The contribution of isobaric interferences on ^187^Os by ^187^Re was smaller than 10^−2^.

**Table 2:** Table 2. Summary of Os isotope ratio mesaurements using laser ablation-MC-ICP-MS and HTR-MC-ICP-MS techniques.

	^186^Os/^188^Os	^187^Os/^188^Os*	^190^Os/^188^Os**	^192^Os/^188^Os**	Re/Os
*Solution nebulization*					
JMC (80 ng/mL)	0.12062±0.00001	0.14033±0.00002	1.97165±0.00006	3.0449±0.0002	<0.00002
*Iridosmines* (*LA-MC-ICP-MS*)					
Sorachi Uryu River, Hokkaido, Japan	0.12525±0.00059	0.12812±0.00039	1.9553±0.0032	2.9440±0.0042	<0.000065
Similkameen River, British Columbia, Canada	0.12471±0.00015	0.12655±0.00010	1.9515±0.0006	2.9420±0.0032	<0.00025
Nevjansk, Ural Mountains, Russia***	0.12058±0.00002	0.12058±0.00004	1.9717±0.0003	3.0544±0.0002	<10^−4^
*Ir–Os NPs* (*HTR-MC-ICP-MS*)					
Sorachi Uryu River, Hokkaido, Japan		0.121±0.013			<10^−2^
Similkameen River, British Columbia, Canada		0.110±0.012			<10^−2^
Nevjansk, Ural Mountains, Russia		0.122±0.020			<10^−2^

Errors are 2σ_m_. Mass discrimination effect was corrected by normalizing ^189^Os/^188^Os being 1.216 [33].*No correction for the isobaric interferences on ^187^Os by ^187^Re was made. **No corrections for the isobaric interferences on ^190^Os and ^192^Os by ^190^Pt and ^192^Pt were made. ***Literature values [17]

### Size distribution analysis of Ir–Os bearing NPs

Sizes of the NPs were calculated based on the signal intensities of single events of the samples and those of the standard size NPs. Generally, size calibration is carried out using standard NPs having the same chemical composition as the unknown samples.^3,9)^ However, there are no commercially available size standard NPs available for the Ir–Os alloy. Hence, the size analysis was based on a calibration using the commercially available Au NP (sizes 60±6 nm) from nanoComposix (San Diego, CA, USA) under the assumption that the elemental sensitivity of Au was identical to that for Ir and Os. The transmission efficiencies of Au and Pt (from sample to ion detector) were 0.058±0.006% and 0.052±0.006%, respectively, suggesting that there is a small difference in the transmission efficiencies between Au and Pt when the same system setup and operational settings are used. The similarity in transmission efficiencies of Au and Pt can also be demonstrated when conducting size calibration of 50 nm Pt NPs using the 50 nm Au NPs (47.8±1.8 nm: nanoComposix). The resulting size of the 50 nm Pt NPs was 50.0±4.9 nm, in good agreement with the data sheet provided by the manufacturer.

Another important assumption is that there were no variations in the Ir/Os abundance ratios among the produced NPs. Due to the limited mass dispersion of the high-gain ion counters, it was not possible to simultaneously determine the ^185^Re/^189^Os, ^187^(Re+Os)/^189^Os, and ^191^Ir/^189^Os ratios. Our conventional LA-MC-ICP-MS analysis on the Ir–Os bearing minerals samples showed that Ir/Os abundance ratios varied from 1.5 to 1.7 with the analysis spot, suggesting that Ir and Os is distributed heterogeneously within a single grain. The effect of the variations in the Ir/Os ratio on the measured sizes of the NPs was smaller than 5%. This is much smaller than the variations in the size distribution of the resulting Ir–Os bearing NPs, so we assumed that Ir/Os ratio for the NPs would be 1.6.

**Figure 3:**
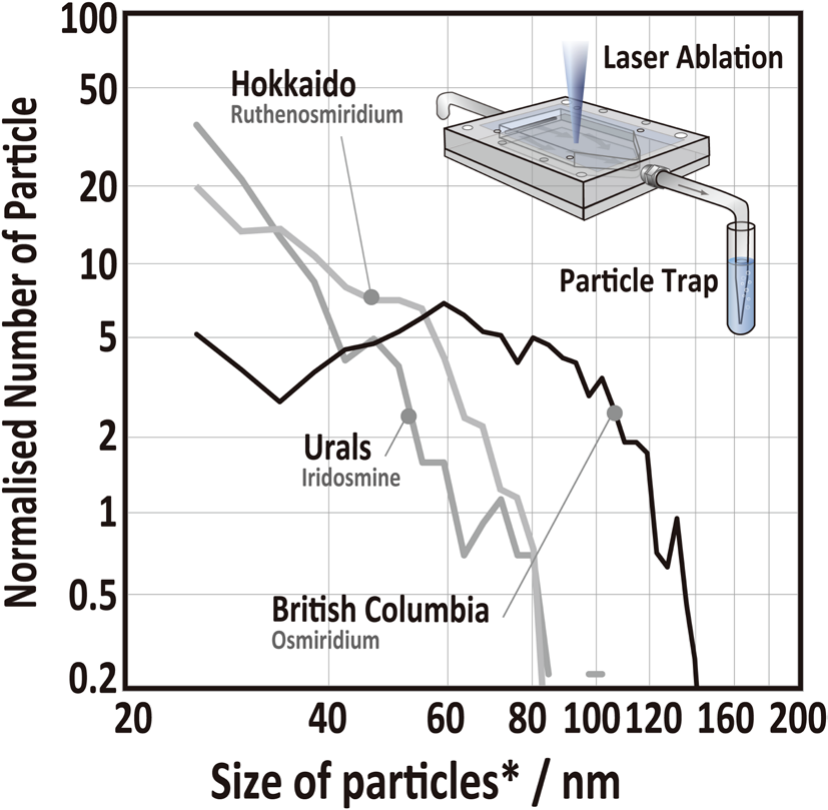
Fig. 3. Measured size distribution of Os–Ir bearing NPs produced by femtosecond laser ablation technique. Sizes of the particles were calibrated by Au size standard NPs on the assumption that elemental sensitivities of Os and Ir were identical to that of Au.

Figure 3 illustrates the measured size distribution of the laser induced NPs. The total number of particles, obtained from three Ir–Os bearing minerals, having different totals numbere of particles, were normalized to 100 in order to obtain a better comparison. The resulting size distribution for the Hokkaido and Urals samples showed that the frequency of the particles decrease with increasing size. A nearly linear correlation of the number with the diameter (Fig. 3: double logarithmic scale) suggests that the resulting particle size distribution reflects Gaudin–Schumann or Roshin–Rammler distributions.^41,42)^ The largest particles were <80 nm for both the Hokkaido and Urals samples. On the other hand, the British Columbia samples showed a unique distribution pattern, with the highest sizes at about 60 nm. In addition, the size distribution data for the British Columbia sample showed the presence of large-sized particles being >80 nm. This contrasts with the size distributions for the Hokkaido and Urals samples, and this difference could be attributed to the crystallographical features of the sample. As shown in Fig. 2, ruthenosmiridium from Hokkaido and iridosmine from the Urals have well crystalline morphologies, whereas there are vacancies in the osmiridium sample collected from British Columbia, suggesting that the small grains may have undergone agglomeration in the sample. This implies that the laser induced shockwave causes the release of small pieces or fragments from the osmiridium samples during the laser ablation procedure.

Under the assumption that the overall transmission efficiency of Os is identical to that of Au, the particle size detection limits for Os achieved by the present system setup is about 10 nm. This is twice as large as that for other elements, such as Au, or Ag. The poor detection limits for Os are mainly due to the low isotopic abundance of the monitored isotopes (^188^Os: 13.3%^33)^). If a higher isotopic abundance of Os (*e.g.*, ^192^Os) was monitored, the resulting size detection limits could have been improved.

### ^187^Os/^188^Os ratio analysis using HTR-MC-ICP-MS

The ^187^Os/^188^Os ratio measurements were carried out on three NPs prepared from Ir–Os bearing minerals (Hokkaido, British Columbia, and Urals) using the HTR-MC-ICP-MS system developed in this study. The total number of particles analysed in this study was 1300 for Hokkaido, 1200 for British Columbia, and 160 for the Urals. The number of particles for the Urals was significantly low, simply because of the smaller size of the Urals iridosmine sample (100×300 μm). The resulting ^187^Os/^188^Os ratios are listed in Table 2, together with the Os isotope ratio data obtained by the conventional LA-MC-ICP-MS technique described in the earlier section. In Fig. 3, the measured ^187^Os/^188^Os ratios for the individual grains are plotted against the size of the particles, showing that the variations in the measured ^187^Os/^188^Os ratios became smaller with increasing particle size. This suggests that the precision of the isotope ratio measurements can be controlled by the counting statistics on the analytes, which is consistent with our previous report.^41)^ To calculate the ^187^Os/^188^Os ratios for each sample, the weighted mean based on the counting statistics on both the ^187^Os and ^188^Os were employed. The ^187^Os/^188^Os ratios obtained here were 0.121±0.013 (*N*=1300, 2σ_m_) for Hokkaido; 0.110±0.012 (*N*=1200, 2σ_m_) for British Columbia; and 0.122±0.020 (*N*=160, 2σ_m_) for the Urals. These results are in agreement with the ratios obtained by the conventional LA-ICP-MS technique obtained in this study: 0.12812±0.00039 (2σ_m_) for the Hokkaido; 0.12655±0.00010 (2σ_m_) for the British Columbia; and 0.12058±0.00004 (2σ_m_) for the Urals.

**Figure 4:**
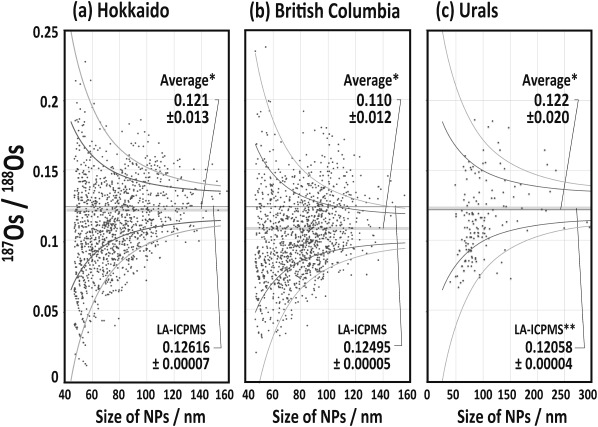
Fig. 4. Measured ^187^Os/^188^Os ratios obtained for NPs prepared from the Ir–Os bearing minerals using the HTR-MC-ICP-MS technique. *Weighted mean. Errors are 2σ_m_. **Reported values [17].

The resulting precision of the ^187^Os/^188^Os ratio measurements were 10–20%, slightly poorer than those achieved in the ^195^Pt/^194^Pt ratio measurements from NPs of similar sizes.^43)^ In the case of the ^195^Pt/^194^Pt ratio measurements, a precision of better than 10% can be achieved from the Pt NPs with sizes being >50 nm. For the ^187^Os/^188^Os ratio measurements, the deterioration of the precision of the measurements can be attributed to a larger contribution of counting statistics originating from the smaller isotopic abundances for both ^187^Os (1.6%) and ^188^Os (13%). The possible variations in the ^187^Os/^188^Os ratios estimated, based on the counting statistics (±1σ and ±2σ) are also shown as grey-coloured curves in Fig. 4. Most of the data points for ^187^Os/^188^Os ratios were plotted within the range of ±2σ variations, suggesting that the precision of the ^187^Os/^188^Os ratio measurements was principally controlled by the counting statistics of the signals. If that is the case, improvements in the transmission efficiency of the mass spectrometer (*i.e.*, sensitivity) is the most important factor in terms of obtaining better precision in the isotope ratio measurements from the NPs. Based on the results gathered in this study, a variation of ^187^Os/^188^Os was observed for minerals of different chemical compositions. Variations in the ^187^Os/^188^Os ratios in excess of 50% may possibly include information that reflects different nucleosynthetic processes (*e.g.*, rapid, slow, or spallation processes). Therefore, the HTR-MC-ICP-MS system developed in this study could be applied to understanding the origin of the heavy elements in small metal nuggets or NPs in meteorites.^21,25,26,44,45)^

## CONCLUSION

^185^Re/^188^Os, ^187^Os/^188^Os, and ^189^Os/^188^Os isotope ratio measurements were made on a series of Ir–Os NPs using a high-time resolution-MC-ICP-MS (HTR-MC-ICP-MS) system. The Ir–Os bearing NPs were produced by laser ablation using a UV femtosecond laser (wavelength of 260 nm, and a pulse duration of 230 fs). The size of the resulting NPs was calibrated with Au NPs of known sizes based on two assumptions: (a) the transmission of Os was identical to Ir and Au, and (b) there were no variations in the Os/Ir ratio among the individual NPs. The resulting sizes of the laser induced NPs ranging from 20–160 nm was within the range of the present HTR-MC-ICP-MS technique.

The resulting precisions of the ^187^Os/^188^Os ratio measurements were 10–20% (2σ_m_), which were principally controlled by the counting statistics of the ^187^Os and ^188^Os signals. Despite the lower precision values obtained for the isotope ratio measurements, the measured ^187^Os/^188^Os ratios were in good agreement with those obtained by the conventional laser ablation-MC-ICP-MS. The precision of elemental or isotope ratio measurements can be further improved using an ICP-MS system with an enhanced sensitivity.

The data reported herein demonstrate that the HTR-MC-ICP-MS system has the potential for being an effective tool for collecting elemental and isotopic ratio data from NPs. With the elemental data obtained from individual NPs, quality control of the industrial raw materials for cosmetics, catalysis, electronic devices, or semiconductor materials, can be made with a high analysis throughput. Moreover, the sensitive detection of NPs from biological tissue samples can provide key information concerning the toxicity of the metal NPs. More importantly, Pb isotope ratios for fine airborne particles, including PM0.1, can be used for both the fundamental studies and for understanding the transport mechanism of particulate matter.

## Author Contributions

T.H. conceived the basis for the methodology, carried out the LA-MC-ICP-MS experiments, and compiled all data and wrote the manuscript. S.Y. carried out both the system setup and isotopic analysis using the HTR-MC-ICP-MS system developed in this study. M.I. conducted the synthesis of the nanoparticles of various materials. T.S. developed the in-house data reduction software for the HTR-MC-ICP-MS system developed in this study.

## Notes

The authors declare no competing financial interest.
